# Synaptic Dysfunction in Alzheimer’s Disease and Glaucoma: From Common Degenerative Mechanisms Toward Neuroprotection

**DOI:** 10.3389/fncel.2017.00053

**Published:** 2017-02-27

**Authors:** Chiara Criscuolo, Carlotta Fabiani, Elisa Cerri, Luciano Domenici

**Affiliations:** ^1^Neuroscience Institute of the National Council of Research (CNR)Pisa, Italy; ^2^Department of Biotechnological and Applied Clinical Sciences (DISCAB), University of L’AquilaL’Aquila, Italy

**Keywords:** beta amyloid, tau, synaptic dysfunction, visual system impairment, brain-derived neurotrophic factor

## Abstract

Alzheimer’s disease (AD) and glaucoma are two distinct multifactorial neurodegenerative diseases, primarily affecting the elderly. Common pathophysiological mechanisms have been elucidated in the past decades. First of all both diseases are progressive, with AD leading to dementia and glaucoma inducing blindness. Pathologically, they all feature synaptic dysfunction with changes of neuronal circuitry, progressive accumulation of protein aggregates such as the beta amyloid (Aβ) and intracellular microtubule inclusions containing hyperphosphorylated tau, which belongs to microtubule associated protein family. During an early phase of degeneration, both diseases are characterized by synaptic dysfunction and changes of mitogen-activated protein kinases (MAPK). Common degenerative mechanisms underlying both diseases are discussed here, along with recent results on the potential use of the visual system as a biomarker for diagnosis and progression of AD. Common neuropathological changes and mechanisms in AD and glaucoma have facilitated the transfer of therapeutic strategies between diseases. In particular, we discuss past and present evidence for neuroprotective effects of brain-derived neurotrophic factor (BDNF).

## Introduction

Alzheimer’s disease (AD) is a neurodegenerative progressive disease of the elderly leading to dementia. The world Alzheimer report (Alzheimer’s Disease International) of 2015 indicated that 46.8 million people worldwide are living with dementia; this number is expected to double every 20 years (Reitz and Mayeux, 2014). There are two forms of AD.

1. Early onset familial alzheimer disease (eFAD). Abnormalities of the amyloid precursor protein (APP) that render it more amyloidogenic, or defects of processing normal APP cause genetic forms of AD. The literature estimates that eFAD accounts for approximately 2% of all people with dementia (approximately 3%–5% of all Alzheimer cases; Mayeux and Stern, 2012; Tanzi, 2012). In these patients, autosomal dominant AD usually develops before age 65 due to mutations of the *APP* gene on chromosome 21 or the presenilin 1 and 2 genes (*PSEN1* and *PSEN2*) on chromosomes 14 and 1, respectively.
2. Sporadic AD (SAD, Late-Onset). SAD is very common in the elderly (approximately 70% of patients with dementia are attributed to SAD; Reitz and Mayeux, 2014). The cause of SAD is unknown. The vast majority of SAD is not genetically inherited although some genes such as the *APOE* may act as a major risk factor (Liu et al., 2013).

Vascular diseases such as hypertension and brain ischemia (Pluta et al., 2013; Origlia et al., 2014), diabetes (Zhao et al., 2008; Adzovic and Domenici, 2014), traumatic brain injury (Van Den Heuvel et al., 2007) and mood disorders (Tsuno and Homma, 2009) represent risk factors for SAD; the most important risk factor for SAD is aging. Neuropathological changes of AD include classical hallmarks such as the senile plaques formed by beta amyloid (Aβ), neurofibrillary tangles (NFTs) and dystrophic neurites containing hyperphosphorylated tau (Serrano-Pozo et al., 2011). Histopathological findings showed that the entorhinal cortex and hippocampus are affected during the earliest phases of AD (Braak and Braak, 1991, 1993). Recent studies have highlighted an apparent dichotomy between the progress of histopathological findings in different brain areas and the occurrence of visual dysfunctions in AD (Moschos et al., 2012; Sivak, 2013; Yamasaki et al., 2016). More than 60% of people with AD have a decline in one or more visual function(s). AD causes vision impairment by affecting the eye (Graw, 2015) and by deterioration of visual functions, from retina to visual cortex. In particular, clinical studies support a link between cognitive performance and visual dysfunction even at an early stage of AD; the gradual loss of memory is frequently accompanied by alteration of visuospatial function in animal models and AD patients (Rizzo et al., 2000; Crow et al., 2003). Recently, we showed an impairment of the visual responses arising from the magnocellular streams of visual processing (Sartucci et al., 2010), suggesting that large retinal ganglion cells (RGCs) are primarily affected in AD. Often, retinal involvement is an early occurrence in AD (Sivak, 2013) as also suggested by results obtained by the use of ocular imaging techniques such as the optical coherence tomography (Moschos et al., 2012).

Glaucoma is a group of eye disorders, currently recognized to be multifactorial and progressive, leading to reduction in vision and eventual blindness. Usually glaucoma affects the older population. Over 60 million people worldwide were estimated to be affected with glaucoma, and bilateral blindness from the disease was estimated to be present in 4.5 million people with glaucoma (Quigley and Broman, 2006). Glaucoma is characterized by the progressive degeneration of RGCs till cell death, optic nerve (ON) atrophy, impairment of visual function with visual field defects and finally, loss of neurons in the lateral geniculate nucleus and primary visual cortex. A generally accepted theory suggests an initial insult to the axons of RGCs in the ON head region (Quigley, 2011). Several types of glaucoma are known; these can be divided in primary and secondary. Primary open-angle glaucoma (POAG) is considered to be the most common subtype of glaucoma. There are two main types of POAG: one that occurs with an intraocular pressure (IOP) higher than normal and represents 60%–70% of the total POAG (Coleman and Brigatti, 2001; Quigley and Broman, 2006) and the other occurring with a normal or lower IOP. Thus, ocular hypertension represents the major risk factor for glaucoma onset and progression. In the presence of ocular hypertension, there is no obvious damage to the RGCs and ON or evidence of visual field changes. However, we recently showed that retinal responses to patterned visual stimuli (pattern electroretinogram, P-ERG) together with Brn3 (POU-domain transcription factor) expressed in RGCs are altered during ocular hypertension in a murine model of glaucoma (Domenici et al., 2014). It is reasonable to think that ocular hypertension applies some stress to RGCs and their circuitry during a phase preceding the degeneration of RGCs and ON atrophy.

## Common Features Between AD and Glaucoma

Both diseases affect older populations and are neurodegenerative, chronic and progressive leading to irreversible cell death. AD and glaucoma are thought to share, at least in part, some common features such as the Aβ accumulation/aggregation, tau aggregation and hyperphosphorylation. Both diseases are characterized by early changes of neuronal circuitry and phosphorylation of mitogen-activated protein kinases (MAPK) followed by inflammatory process, glial reaction, reactive oxygen species production, oxidative stress and mitochondrial abnormalities, propagation of neurodegenerative processes leading to cell death. Both diseases are characterized by common features such as synaptic dysfunction and neuronal cell death at the level of the inner retina (Sartucci et al., 2010; Sivak, 2013). Taken together, all these observations suggest similar degenerative mechanisms between AD and glaucoma. Glaucoma is recognized as a disease frequently associated with AD and aging (Martinez et al., 1982; Chandra et al., 1986; Tamura et al., 2006; Tsolaki et al., 2011; Jefferis et al., 2013; Elyashiv et al., 2014). Conflicting data have been reported among the different studies carried out to compare AD frequency in glaucoma patients (Kessing et al., 2007; Yochim et al., 2012). Thus, a clear epidemiologic relationship between glaucoma and AD remains elusive.

## Amyloid-Dependent Mechanisms in AD and Glaucoma

Senile plaques in AD comprise aggregates of Aβ filaments, dystrophic neurites and mitochondrial abnormalities (Hirai et al., 2001; Serrano-Pozo et al., 2011). Aβ peptides start to be generated in considerable amounts by the cleavage of APP due to sequential activation of β- and presenilin catalytic site of γ-secretases. Aβ can be found in different compositions of monomers, oligomers, or fibrils (Stromer and Serpell, 2005); in particular, increasing Aβ tends to form monomers which aggregate into oligomers, prefibrillar assemblies (protofibrils) and amyloid fibrils in a concentration-dependent manner. Toxic Aβ peptides are formed by 36–43 amino acids; the 42 amino acid peptide (Aβ42) is one of the most neurotoxic amyloidogenic fragment and represents the chief component of senile plaques. Increasing Aβ level tends to form oligomers of different length and composition (Stromer and Serpell, 2005), which are toxic for neuronal cells (Origlia et al., 2008). In particular, Aβ oligomeric extracts from cerebral cortex of AD patients (Shankar et al., 2008) and synthetic Aβ formed by dimers and trimers (Origlia et al., 2008, 2009) are capable of inhibiting long term synaptic plasticity in the form of long term potentiation (LTP), which is involved in learning/memory in hippocampus and parahippocampal cortices (Nabavi et al., 2014); this represents an early step in the disease progression. Oligomeric Aβ inhibits LTP through phosphorylation of p38 MAPK (Criscuolo et al., 2014). Increasing synthetic Aβ concentration affects synaptic transmission, AMPA current (Origlia et al., 2010). Thus, accumulation of extracellular Aβ is likely to result in progressive synaptic dysfunctions and cognitive impairment (Selkoe, 2002). Increasing Aβ induces phosphorylation of MAPKs in neuronal and non-neuronal cells along with the induction of pro-inflammatory cytokines, such as the IL-β (Origlia et al., 2010). Activation of receptors such as the receptor for advanced glycation end products (RAGE) by Aβ accounts for progress of synaptic dysfunction, development of inflammatory and, possibly, oxidative processes, leading cells to degenerate (Origlia et al., 2010).

The death of the RGCs in glaucoma is preceded by a remodeling of retinal circuitry, RGC dendritic arbor and axonal atrophy (Jakobs et al., 2005). Although it is not yet clear what initiates the death of RGCs in glaucoma, recent experimental evidence indicates that functional alterations caused by impairment of synapses (Della Santina et al., 2013) precede the degeneration of RGCs. A possible interpretation would be that the impairment of synapses is followed by consequences for RGC viability. Similarly to AD, enhanced retinal levels of soluble Aβ may act by impairing the synaptic circuitry and retrograde trafficking of neurotrophic factors in the ON axons (Poon et al., 2011; Gupta et al., 2014). Interestingly, high IOP, which characterizes an early reversible phase of retinal degeneration, leads to Aβ induction (McKinnon et al., 2002). The hypothesis can be advanced that stressor stimuli such as the high IOP in glaucoma may cause accumulation of Aβ in the retina, contributing to synaptic progressive dysfunction in the inner retina and impairment of visual responses. However, whether low amounts of Aβ in the form of oligomers result in synaptic toxicity with detriment of visual retinal responses during ocular hypertension is still lacking. Concerning cell death, progressive degeneration of RGCs is associated with increased production of Aβ (McKinnon et al., 2002; Guo et al., 2007). Hence, it is assumed that Aβ leads not only to neuronal cell impairment in AD but also to retinal cell impairment and degeneration in glaucoma in general. Yan et al. (1996) showed that the RAGE activated by Aβ is able to induce neuronal toxicity; it is known that oligomeric Aβ induces phosphorylation of p38 MAPK through RAGE activation during an early phase of degeneration in AD (Origlia et al., 2008). Interestingly, hyperphosphorylation of p38 MAPK characterizes the degeneration of RGCs in glaucoma, mainly during an early phase with high IOP and synaptic dysfunction (Fabiani et al., 2016), whether RAGE is involved in the mechanisms of glaucoma onset and progression is still unanswered.

## Tau-Dependent Mechanisms in AD and Glaucoma

The tau protein is expressed from the gene known as microtubule associated protein tau (MAPT) on chromosome 17. Tau is highly expressed in neurons and is abundant in axons (Lee et al., 2001). Tau facilitates assembly and the stabilization of microtubule polymers (Cleveland et al., 1977; Caceres and Kosik, 1990), modulating microtubule dynamics. Thus, under physiological conditions tau is mainly expressed within neurons. Hyperphosphorylated, insoluble and filamentous tau proteins were shown to be the main component of NFTs, a pathological hallmark of AD and other tauopathies (Lee et al., 2001). NFTs accumulate inside the cells, disrupting the intracellular transport system. Cytoskeletal changes are visible as dystrophic neurites, pre-tangles, NFTs in the cell bodies of affected neurons in AD (Iqbal et al., 1984). Interestingly, phosphorylation of tau potentiates MAPK activation similarly to Aβ and tau is one of p38 MAPK substrates (Corrêa and Eales, 2012). Studies on cell viability have shown that misfolding of tau leads to the aggregation of tau and the appearance of toxic tau species in the extracellular space (Gómez-Ramos et al., 2006, 2008). The endogenous intracellular tau may be released as aggregates to the extracellular space upon neuron degeneration (Gómez-Ramos et al., 2006). Extracellular tau could be toxic by increasing intracellular calcium into neighboring neurons (Gómez-Ramos et al., 2008). The presence of extracellular tau can be due to other causes, for example exocytosis; the N-terminal region of tau seems to be required for its secretion (Kim et al., 2010). Tau can also be released into the extracellular space, as oligomers (Saman et al., 2012). Indeed, neuronal toxicity may be caused by tau aggregates, even small and soluble aggregates in the form of oligomers, which have been identified in AD brains (Sahara et al., 2008). Recently, it has been shown that oligomeric extracellular tau is able to interact with cell receptors resulting in synaptic dysfunction and signaling propagation that could contribute to onset of neurodegeneration (Fá et al., 2016). These observations point to the involvement of extracellular tau aggregates as one of the main agent in the neuron-to-neuron propagation of neurofibrillary pathology and progression of toxicity in AD.

Tau was found to be expressed in RGCs and it is involved in RGC axon development and survival (Lieven et al., 2007). In an aged retina there is an increase in the number of RGCs and photoreceptors expressing phosphorylated tau (Leger et al., 2011). In POAG with ocular hypertension, decreased total tau and increased phosphorylated tau was reported (Gupta et al., 2008). Hyperphosphorylation and aggregation of tau were associated *in vivo* with reduced axonal transport in the ON of transgenic mice line expressing human P301S tau transgene (Gasparini et al., 2011; Bull et al., 2012). However, whether intracellular and/or extracellular tau plays a role in glaucoma onset and progression is still an open question.

## New Perspectives on Therapeutic Approach

Increasing lines of evidence suggest that aggregation and accumulation of Aβ and Tau eventually leading to MAPK phosphorylation represent common degenerative mechanisms in both AD and glaucomatous retinal degeneration. Determining the degenerative mechanisms is crucial for development of new therapeutics. The retina, which is affected in both diseases, can be an important brain area where to investigate common mechanisms also in view of new therapeutics. On this ground, a neuroprotective approach based on neurotrophic factors can be considered promising. Neuroprotection by neurotrophic factors was initially investigated for neurodegenerative diseases such as the AD. Evidence suggests that treatments with neurotrophic factors such as the brain-derived neurotrophic factor (BDNF), ciliary neurotrophic factor (CNTF), glial cell line-derived neurotrophic factor (GDNF) increase the survival of neurons in animal models of injury and disease (Alqawlaq et al., 2012). BDNF, together with its receptor tropomyosin receptor kinase B (TrkB), is highly expressed in several brain areas under the control of neuronal activity (Castrén et al., 1992; Pattabiraman et al., 2005). BDNF acts by binding to the TrkB, activating downstream pathways including the MAPK, phosphatidylinositol kinase (PI3K) and phospholipase C-γ (PLC-γ) signaling cascades (Kaplan and Miller, 2000). BDNF controls synaptic plasticity and cell survival in the visual system (Liu et al., 2007; Schwartz et al., 2011; Kimura et al., 2016). BDNF appears to provide the highest level of protection by supporting both protective and regenerative functions of RGCs in various models of ON injury and disease (Peinado-Ramón et al., 1996; Weber et al., 2008; Parrilla-Reverter et al., 2009). BDNF is locally produced by retinal cells in the ganglion cell and inner nuclear layers (Perez and Caminos, 1995); its TrkB receptor is expressed in RGCs, amacrine and Müller cells (Perez and Caminos, 1995; Cellerino and Kohler, 1997; Wahlin et al., 2001).

BDNF level is reduced in the glaucomatous retina (Pease et al., 2000; Quigley et al., 2000; Fabiani et al., 2016) as well as in several brain areas of AD (Connor et al., 1997; Michalski and Fahnestock, 2003; Peng et al., 2005), thus contributing to advancing the hypothesis that a scarce availability of BDNF renders neurons more vulnerable. In addition, BDNF is considered a peripheral marker of neurodegeneration. BDNF in the tears and/or blood is used for detection and assessment of neurodegenerative processes in POAG (Ghaffariyeh et al., 2009, 2011). The interpretation of the results obtained on BDNF level in blood (serum/plasma) of AD patients is more complex and contradictory (Komulainen et al., 2008; Faria et al., 2014). The idea is that cognitive deficits in AD are related to change of BDNF blood level as well as that of other neurotrophic factors such as the nerve growth factor and GDNF in blood (Budni et al., 2015). Interestingly, Yasutake et al. (2006) showed that there is a decline in blood BDNF once the disease has progressed to severe level. Thus, BDNF level in blood represents a marker of cognitive dysfunction and progress of neurodegeneration in AD and other neurodegenerative diseases such as Parkinson’s disease (Scalzo et al., 2010) and vascular dementia (Yasutake et al., 2006).

BDNF is important for survival and plasticity of the RGCs in models of ON injury and disease (Peinado-Ramón et al., 1996; Weber et al., 2008; Parrilla-Reverter et al., 2009). BDNF delivery into the entorhinal cortex and hippocampus is able to ameliorate cognitive deficits in aging and experimental AD models (Nagahara et al., 2009). Thus, the reported results make BDNF a good candidate to drive full neuroprotective and repair strategies in neurodegenerative diseases, including glaucoma and AD. However, the therapeutic approach based on BDNF is promising if the limits imposed by complex pharmacokinetic of high molecular weight proteins (for example BDNF low propensity to pass blood-brain and blood-ocular barriers following systemic treatment) are definitely defeated. Recently, we showed that BDNF topical eye treatment in the form of collyrium was able to increase the retinal level of BDNF up to rescue visual responses in a murine model of glaucoma (Domenici et al., 2014). Moreover, the intranasal delivery of proteins has recently emerged as a non-invasive and effective method to target high molecular weight proteins such as the BDNF to several brain areas (Alcalá-Barraza et al., 2010; Dhuria et al., 2010). Thus, BDNF non-invasive treatments represent a promising feasible therapeutic strategy to preserve neuronal function and diminish cell vulnerability in neurodegenerative diseases such as the AD and glaucoma. Although at present it is unclear how BDNF concentrations vary *in vivo* the use of high BDNF doses should be avoided to circumvent potential undesired effects such as the proconvulsant effects (Heinrich et al., 2011; Gu et al., 2015) and neovascularization (Lam et al., 2011). Additional therapeutic strategies based on BDNF have been used; these consist of gene delivery (Nagahara et al., 2013), transplantation of BDNF-expressing cell grafts (Kurozumi et al., 2005), TrkB agonists (Hu et al., 2010; Devi and Ohno, 2012; Gu et al., 2015).

## Conclusion

We reported that several common disease features appear in the AD and glaucoma. Based on common disease features that have been described here, several opportunities exist to develop common therapeutic strategies. A successful example involves neuroprotection by BDNF.

## Author Contributions

All authors listed, have made substantial, direct and intellectual contribution to the work, and approved its final version of the manuscript. LD conceived the review focus, conducted literature review and finalized the manuscript. CC summarized and finalized the manuscript. CF conducted literature review and finalized the manuscript. EC conducted literature review and finalized the manuscript.

## Conflict of Interest Statement

The authors declare that the research was conducted in the absence of any commercial or financial relationships that could be construed as a potential conflict of interest.
